# Pulsed ultraviolet (PUV) disinfection of artificially contaminated seawater seeded with high levels of pathogen disease indicators as an alternative for the shellfish industry depuration systems

**DOI:** 10.1007/s11356-023-27286-6

**Published:** 2023-05-08

**Authors:** Gustavo Waltzer Fehrenbach, Emma Murphy, Robert Pogue, Frank Carter, Eoghan Clifford, Ian Major, Neil Rowan

**Affiliations:** 1 Materials Research Institute, Technological University of the Shannon – Midlands Campus, N37 HD68, Athlone, Ireland; 2 LIFE – Health and Biosciences Research Institute, Technological University of the Shannon – Midwest Campus, V94 EC5T, Limerick, Ireland; 3 Post-Graduate Program in Genomic Sciences and Biotechnology, Catholic University of Brasilia, 71966-700, Brasilia, Brazil; 4 Center for Sustainable Disinfection and Sterilization, Bioscience Research Institute, Technological University of the Shannon – Midlands Campus, N37 F6D7, Athlone, Ireland; 5 Coney Island Shellfish Ltd., F91 YH56, Sligo, Ireland; 6 School of Engineering, National University of Ireland Galway, H91 HX31, Galway, Ireland; 7 Ryan Institute, National University of Ireland Galway, H91 HX31, Galway, Ireland

**Keywords:** Disinfection, Foodborne pathogens, Seafood, Depuration, Ultraviolet

## Abstract

The increase in pathogen levels in seawater threatens the safety of entire aquatic ecosystems. Foodborne pathogens can potentially accumulate in shellfish, especially in filter feeders such as bivalves, requiring an efficient depuration process before consumption. Alternative approaches to promote a cost-efficient purge at depuration plants are urgently needed. A small prototype pulsed ultraviolet (PUV) light recirculation system was designed, and its depuration potential was tested in a seawater matrix artificially contaminated with high levels of microbial pathogens *Escherichia coli*, *Staphylococcus aureus*, *Salmonella typhimurium*, *Bacillus cereus* and *Candida albicans*. The analysis of treatment parameters including voltage, number of pulses and duration of treatment was performed to ensure the highest reduction in contaminant levels. Optimal PUV disinfection was attained at 60 pulses/min at 1 kV for 10 min (a UV output of 12.9 J/cm^2^). All reductions were statistically significant, and the greatest was observed for *S. aureus* (5.63 log_10_), followed by *C. albicans* (5.15 log_10_), *S. typhimurium* (5 log_10_), *B. cereus* (4.59 log_10_) and *E. coli* (4.55 log_10_). PUV treatment disrupted the pathogen DNA with the result that *S. aureus*, *C. albicans* and *S. typhimurium* were not detectable by PCR. Regulations were reviewed to address the applicability of PUV treatment as a promising alternative to assist in the reduction of microbial pathogens at depuration plants due to its high efficiency, short treatment period, high UV dose and recirculation system as currently employed in shellfish depuration plants.

## Introduction

Seafood is an important food source and includes finfish, crustaceans, echinoderms and molluscs obtained from fresh and saltwater. Seafood represents approximately 7% of the global food market and is projected to reach a value of $336 billion US dollars by 2025 (Shahbandeh, 2021). Global seafood consumption has increased at an average rate of 3% a year from 1961 to 2019, almost twice the annual world population growth for the same period (FAO, 2022). Consumption per capita grew from 9 kg in 1961 to 20.2 kg in 2020, and the seafood production chain evolved to meet the increased demand. One of the main changes was the rapid development of aquaculture systems with the use of technology such as automated feeding process and traceability of development to maximize yields and increase sustainability, expanding production from 19 million tonnes in 1950 to 179 million tonnes in 2018 (FAO, 2016). Shellfish is a seafood widely cultured in sea-based systems, relying on suitable water and environmental conditions. Bivalves are the most abundantly produced shellfish in Europe and will be referred to in this research. As a result of their filter-feeding mechanism, bivalves are susceptible to water impurities, which may lead to the accumulation of high levels of microbial pathogens (Martinez-Albores et al. 2020). Its filter physiology will also purge pathogens if placed in uncontaminated water and is often realized in depuration plants to purge it to levels within safe limits (EC, 2019).

**Fig. 1:**
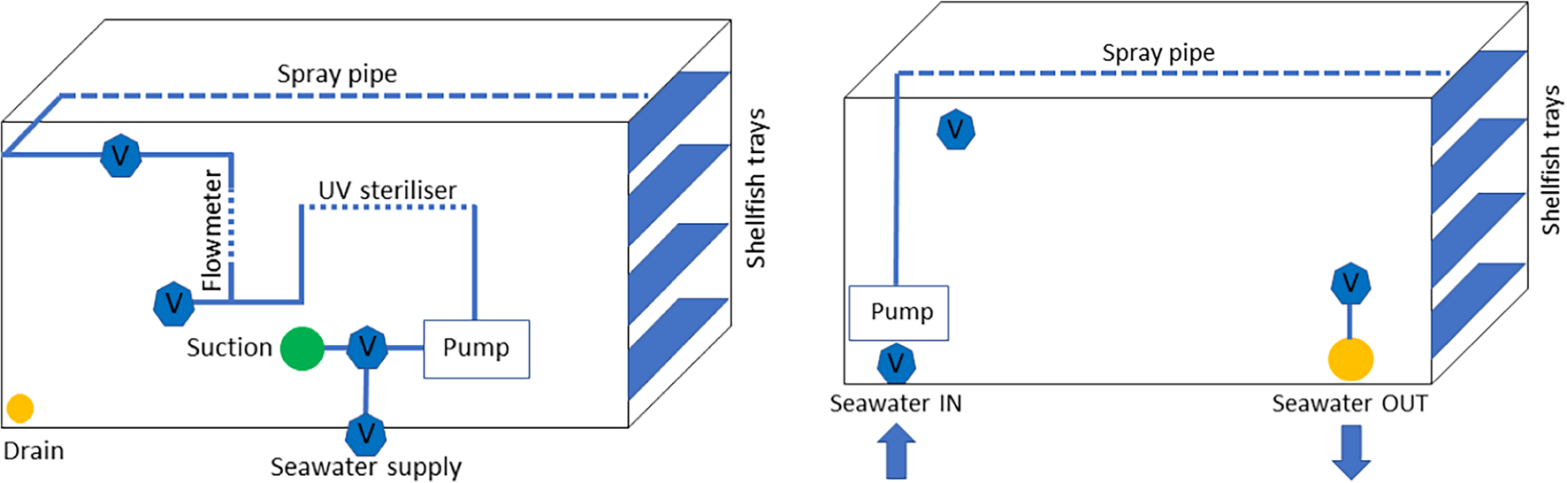
Shellfish depuration system. **a** Recirculation system. **b** Flow-through. V: valves

After harvesting, the live bivalve is washed/cleaned in a processing plant to remove mud and debris, and graded by weight, a process whereby the underweight bivalve is returned to cages in water until achieving the commercial weight. The product is then transported to a depuration facility where it will remain for the depuration period. Seawater is mainly treated on recirculation and flow-through systems in large tanks of usually 1–3 m^3^ and often pre-treated before usage due to the presence of contaminants. The type of depuration system deployed depends on factors such as availability and the average weight of the bivalve treated. Water quality is assessed by the presence of contaminants, phytoplankton concentration and parameters such as temperature, dissolved oxygen, turbidity and salinity. Re-circulation systems rely on water consistency since they can recirculate the same water for at least 24 h (Schneider et al. 2009). Such systems (Fig. 1a) usually consist of a pump that pumps water from the tank, recirculates it through a UV treatment and sprays it on the bivalve seated on trays. Chlorine, iodophors and ozone are also used in the depuration process to deactivate pathogens such as *E. coli* and NOV released from the bivalve and in the pre-treatment of seawater. In flow-through systems (Fig. 1b), seawater is continuously pumped through the depuration system and will discharge fractions of the water and replenish it during the depuration process. A clean and reliable source of seawater is necessary to provide water free of contaminants, and UV or membrane filtration can enhance seawater quality prior to pumping into the depuration system.

The disuse of chlorine treatment is due to several effects on bivalves such as shutting down the filter-feeding activity on oysters and carcinogenic potential of chlorinated metabolites, requiring a post-treatment with vigorous agitation and degassing with thiosulphate (Schneider et al. 2009; Martinez-Albores et al. 2020). Iodophors are substances whereby their bactericidal activity is based on elemental iodine, which penetrate the cell walls and membranes interfering with DNA synthesis. Their efficiency and safety for bivalve’s depuration system have not been widely researched. Ozone is a strong oxidizing agent that inactivates contaminants by attacking the double bonds of organic compounds, supporting its use on lipophilic toxins. It has an increased operation cost and can generate cancerous co-products such as bromates. Of the available techniques, UV disinfection is distinct due to significant reductions in the viability of waterborne pathogens without leaving residuals and is the most widely used method in the UK, the USA and Australia (McLeod et al. 2017) and partially used in China where ozone is also applied (Lee et al. 2008).

The treatment efficacy is due to severe damage to cell structure from the activation of the photoreactive potential of purines and pyrimidines in DNA that triggers the formation of mutations, oxidative stress and production of reactive oxygen species (ROS). These ROS attack nucleotide pools and stress-mediated mutagenesis (Ikehata and Ono, 2011). The current UV systems operate continuously and inactivate pathogens in recirculated water from depuration tanks. They are typically low-pressure mercury UV bulbs working mostly monochromatically from 100 to 400 nm and approximately 40 W. Treatment efficacy results from severe damage to a pathogen’s cell structure. The efficacy of the method may also be reduced when enteric viruses (such as NOV) are present, because higher UV doses and extended retention times are required to reach safety levels. In this case, however, the use of high-power systems with wider wavelengths and UV doses can overcome these challenges, reducing the average time a certain amount of water stays in the depuration plant (hydraulic retention time) and ensuring food safety.

Pulsed ultraviolet (PUV) light treatment is based on high peak power pulses of > 1 kW/cm^2^ generated by xenon lamps and delivered at short intervals (100 ns to 2 ms) within a broad wavelength spectrum ranging from 100 to 1100 nm. Its disinfection efficacy was reported for different pathogens and areas such as the packaging industry (Garvey and Rowan, 2015; Chen et al. 2015), surfaces in an animal laboratory (Li et al. 2020), artificially contaminated wastewater (Fitzhenry et al. 2021), different types of milk (Ansari et al. 2019) and cheese (Ricciardi et al. 2020). PUV is a promising alternative for the food industry as there is no by-product identified for PUV treatment (Rowan, 2019), and its application in food products is authorized by the United States Food and Drug Administration (FDA, 1999). Its high power and UV dose can potentially inactivate pathogens in the shellfish depuration system.

In Europe, the level of contaminants is constantly monitored in designated shellfish production areas which are classified as A, B and C. Bivalve molluscs from class A shall not exceed 230 *Escherichia coli* per 100 g of flesh and intravalvular liquid in 80% of samples collected, considered safe to consume if no other health risk is present. Class B is designated for areas where *E. coli* abundance does not exceed 4600 in 90% of the samples per 100 g of flesh and intravalvular liquid and 46,000 per 100 g of flesh and intravalvular liquid for class C. *E. coli* is still the main parameter for monitoring as most of the shellfish contamination arises from human and animal faeces, which suggests the presence of other contaminants also present in faeces but in lower concentrations. When harvested from class B and C areas, shellfish must be purged in depuration plants. Other contaminants such as paralytic shellfish poison (PSP) and norovirus (NOV) are also periodically monitored, and regulations follow the basic principles of food law to protect human health and consumer interests as set by Regulation (EC) No. 178/2002 (EC, 2002). The Centre for Environment, Fisheries and Aquaculture Science (CEFAS, 2022) recommends a minimum of 42 h to depurate the shellfish from class A and B harvesting areas. Then, it can be commercialized if it is within the limits established for the following parameters: organoleptic characteristics (1), biotoxins (2), *E. coli* (3), *S. typhimurium* (4), norovirus (5) and hepatitis A (6). The first parameter requires an absence of dirt on shells, a normal amount of intravalvular liquid and adequate response to percussion. Biotoxins must be within limits, such as paralytic shellfish poison (< 800 μg/kg), amnesic shellfish poison (< 20 mg of domoic acid/kg), yessotoxins (1 mg/kg) and azaspiracods (160 μg/kg). *Salmonella* should not be detected in 25 g of bivalve tissue (EC, 2004). Hepatitis A and norovirus (NOV) are highly contagious, and there is no consensus or regulations for limits of RNA copies in shellfish which are monitored and controlled in a pro-active way to preserve consumer safety (FSAI et al. 2018).

In this paper, we describe and report the test results from a small prototype pulsed ultraviolet recirculation system for purification of seawater artificially contaminated with high levels of *Escherichia coli*, *Staphylococcus aureus*, *Bacillus cereus*, *Candida albicans* and *Salmonella typhimurium*. For example, *Escherichia coli* and *Salmonella* species were specifically chosen because they are used as index pathogens of faecal contamination for water quality assessment (Holcomb and Stewart, 2020). *Candida albicans* was chosen because it is representative of a clinical yeast occurring in wastewater (Babič et al. 2017). *Staphylococcus aureus* was chosen because it is associated with foodborne intoxications (FSAI, 2011). These organisms are also representative of Gram-positive and Gram-negative pathogens, which allows for verifying the effectiveness of PUV in different cellular structures. The proposed treatment described here was developed to be further tested in and ultimately employed by the shellfish industry at depuration plants to assist in the disinfection from common foodborne pathogens.

## Material and methods

### Pulsed-ultraviolet treatment

**Fig. 2:**
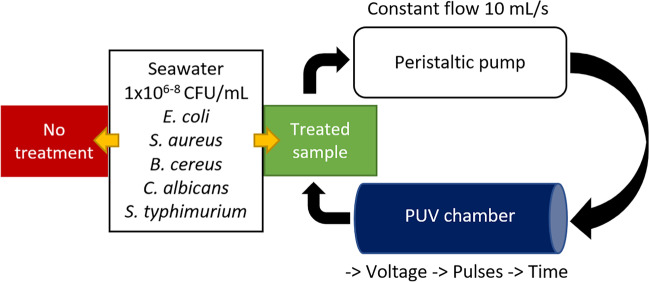
PUV treatment, foodborne pathogens and main parameters associated with the treatment

A small pulsed ultraviolet (PUV) light reactor was built to be further tested in the depuration process of the shellfish industry. The 60-kPa low-pressure xenon-filled flash lamp (Heraeus Noblelight XAP type NL4006) was fixed in stainless steel chamber of 100 mL volume internally walled with glass. The system was powered by a 1000-V scale power source (Samtech Pulsed UV 2018) equipped with an automatic trigger system. Purification was performed in a recirculation regime, and a schematic is presented in Fig. 2. A peristaltic pump (ColeParmer) was employed to recirculate 200 mL of contaminated seawater of salinity of 35 ppt and a temperature of 18 °C at a constant flowrate of 10 mL/s with hydraulic retention time (HRT) of 0.10 s. Using the same inoculum, no treatment (Fig. 2) was performed but not exposed to PUV, and each pathogen was recirculated through the system under the same conditions. The efficacy of PUV treatment was then compared to no treatment.

PUV treatment conditions were selected based on log reduction after treatment at different voltage intensities (V), number of pulses per min and time (min) as presented in Table 1.

**Table 1:** PUV preliminary experiments to identify the best treatment conditions

Treatment	Voltage (V)	Number of pulses per min	Duration (min)
1	500	6	1
2	1000	6	1
3	500	6	10
4	1000	6	10
5	500	60	10
6	1000	60	10

Energy, peak current, peak power, peak admittance and current rise/fall time at 500 and 1000 V are presented in Table 2. Values are available in the PUV manufacturer’s manual. Energy (J) corresponds to release per pulse in the flash lamp. Peak current (A) and power (kW) are the maximum current and power at the peak of pulsed energy, respectively, while peak admittance (S) is a measure of how easily the current will flow through the system and reach the lamp. Current rise/fall corresponds to time (μs) until it reaches the peak current and returns to 0.

**Table 2:** Operating parameters of PUV lamp at 500 and 1000 V

Discharge voltage (V)	Energy per pulse (J)	Peak current (A)	Peak power (kW)	Peak admittance (S)	Current rise/fall time (μs)
500	5	443	175	1.10	12/36
1000	20	1173	985	1.46	7/27

UV output was determined by the product of UV irradiance (mJ/cm^2^), therefore a wavelength < 300 nm, and exposure time (s). The calculations are presented below.

1. Energy output (Eo) was determined by the product of energy per pulse (J or W/s) and pulses per second (0.1 and 1).
2. Energy intensity (Ea) was determined by the division of Eo by the lamp surface area (170 cm^2^ - height: 26 cm and radius: 1 cm).
3. Considering the ratio of energy generated at the UV range (< 300 nm) as 0.18 (Appendix 1), the product of Ea by 0.18 resulted in the energy intensity in the UV region (EaUV).
4. Finally, UV output (J/cm^2^) after complete treatment was calculated by the product of exposure time (s) and EaUV (Table 3).

**Table 3:** UV output (J/cm^2^) at 500 and 1000 V when applying 0.1 and 1 pulses per second for 1 and 10 min

Discharge voltage (V)	Energy per pulse (J)	Pulses per second	Eo (W)	Ea (mW/cm^2^)	EaUV (mW/cm^2^)	Exposure time (s)	UV output (J/cm^2^)
500	5	0.1	0.5	2.941	0.529	60	0.0324
		0.1	0.5	2.941	0.529	600	0.324
		1	5	29.41	5.29	600	3.24
1000	20	0.1	2	11.76	2.11	60	0.129
		0.1	2	11.76	2.11	600	1.29
		1	20	117.6	21.1	600	12.9

**Fig. 3:**
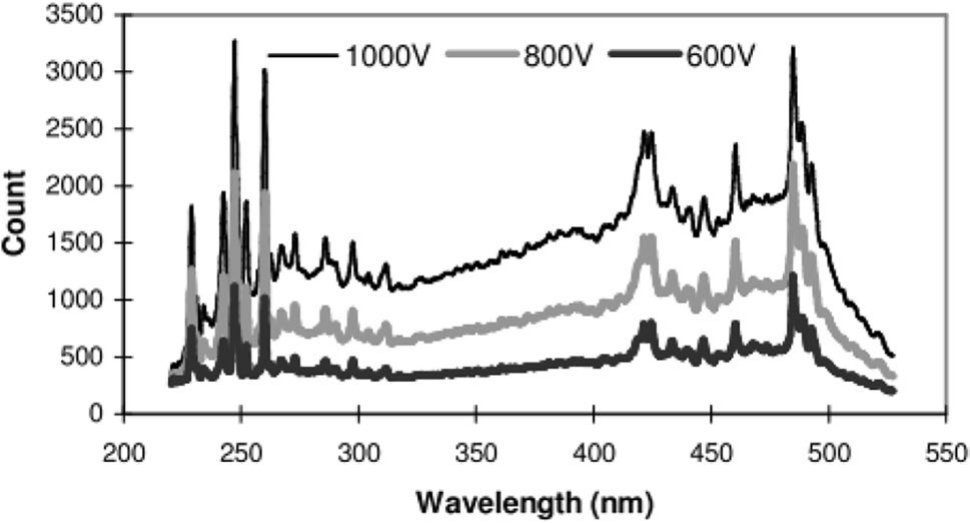
Wavelength spectrum from emitted PUV flash (Samtech Pulsed UV 2018)

The spectrum emitted from the flash lamp is presented in Fig. 3. The voltages of 500 and 1000 V have similar spectra, with high output in the UVC region (220–80 nm) and visible light (430, 460 and 500 nm).

### Foodborne pathogens and culture conditions

**Fig. 4:**
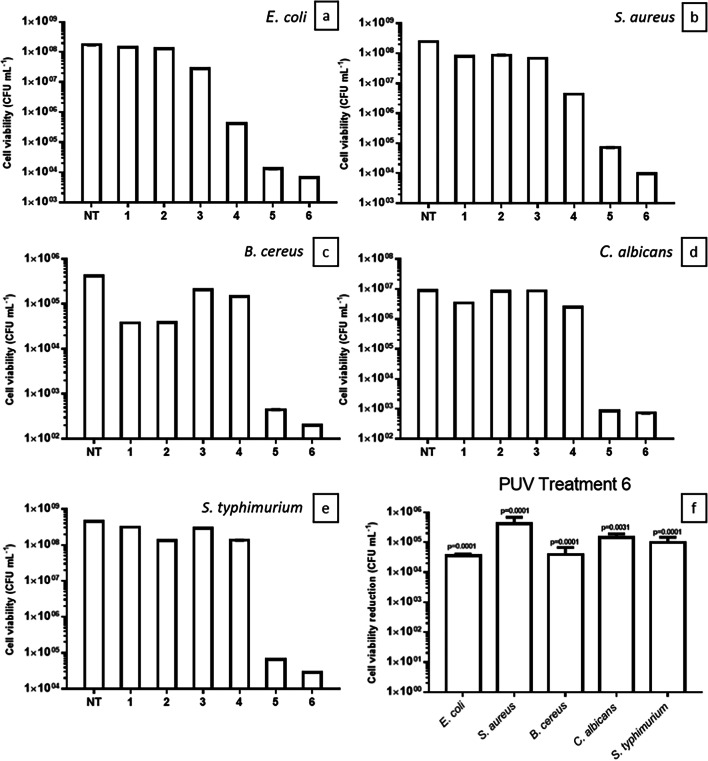
Cell viability (CFU/mL) of *Escherichia coli* (**a**), *Staphylococcus aureus* (**b**), *Bacillus cereus* (**c**), *Candida albicans* (**d**) and *Salmonella typhimurium* (**e**) not treated (NT) and after PUV treatment at different conditions (**f**). CVR of pathogens under optimal conditions identified on repeated treatment 6 (1000 V, 60 pulses/min and 10-min treatment). Standard deviation bars are presented in ± CFU/mL. Statistical significance (*p* < 0.05) was observed in all pathogens treated with PUV at optimal conditions when comparing viabilities after treatment with no treatment levels

Bacterial species detected in shellfish and yeast associated with foodborne illness were selected to simulate a severe contamination of seawater. Contaminant levels in the seafood industry are much lower than the levels tested here; however, this approach was chosen to test the robustness of the PUV method. *Escherichia coli* (ATCC25922), *Staphylococcus aureus* (ATCC29213), *Bacillus cereus* (ATCC11778), *Candida albicans* (ATCC10231) and *Salmonella typhimurium* (IMD121) were selected and cultivated at under specific conditions and with specific media. *E. coli and S. aureus* were cultivated in Luria Bertani (10 g/L peptone, 5 g/L yeast extract and 5 g/L chloride), while *B. cereus*, *C. albicans* and *S. typhimurium* in Brain Heart Infusion (200 g/L calf brain, 250 g/L beef heart, 10 g/L proteose peptone, 5 g/L sodium chloride, 2.5 g/L sodium phosphate, 2 g/L dextrose), Potato Dextrose Broth (200 g/L potato infusion, 20 g/L dextrose) and Tryptic Soy Broth (17 g/L peptone from casein, 3 g/L peptone from soymeal, 2.5 g/L D(+) glucose monohydrate, 5 g/L sodium chloride, 2.5 g/L di-potassium hydrogen phosphate), respectively. Flasks containing media and respective strains were then cultivated in a shaker at 28 °C (*C. albicans*) and 37 °C (*E. coli*, *S. aureus*, *B. cereus*, *S. typhimurium*) to exponential phase and used as inoculum. Seawater was collected at Sligo Bay (54.3432° N, 8.5728° W) and autoclaved for 15 min at 121 °C to be used as a treatment matrix. A salinity of 35 ppt was observed, and pH was 7.6. The spiking was performed at room temperature (18 °C), and strains were treated in separate reactions at 10^6–8^ CFU/mL. Experiments were run as presented in Fig. 2. The PUV system was washed and sanitized with isopropanol 70° several times to ensure complete cleaning. The average initial concentration of pathogens in preliminary assays was 1.8 × 10^8^ ± 1.68 CFU/mL (Fig. 4a–e) and 2 × 10^9^ ± 2.31 CFU/mL (Fig. 4f) when re-testing treatment 6 conditions.

### Cell viability

A spread plate method was used to count the number of viable cells of no treatment (NT) and treated (T) with PUV. Samples were collected, serially diluted, spread onto agar plates and incubated at 28 °C (*C. albicans*) and 37 °C (other pathogens) for 36 and 24 h, respectively. Cell viability reduction (CVR) was calculated as presented in Eq. 1.

$$\textrm{CVR}={\log}_{10}\frac{\textrm{NT}}{\textrm{T}}$$

where NT is no treatment, and T is treated

### DNA extraction

Bacterial genomic DNA was extracted as described on the GenElute kit (Sigma-Aldrich) with modifications. Samples of 2 mL were collected before and after PUV treatment and non-treated used as a positive control. Samples were centrifuged at 13,000 rpm for 2 min, and pellets of *B. cereus*, *S. aureus* and *C. albicans* were pre-treated with 200 μL of lysozyme for 30 min at 37 °C, while *E. coli* and *S. typhimurium* incubated with 20 μL of proteinase K for 30 min at 55 °C. To assist with DNA extraction, *C. albicans* was sonicated (35 W/40 °C) for 20 min prior to incubation with lysozyme. Proteinase K was then added to Gram-positive species, and 200 μL of lysis solution C was added to all strains and incubated for 10 min at 55 °C. The subsequent steps of column preparation, binding and washing steps were performed as described in the kit. DNA retained in the column was eluted with elution solution (10 mM Tris-HCl, 0.5 mM EDTA, pH 9.0) and stored at − 20 °C.

### Amplification conditions and gel electrophoresis

Polymerase chain reaction (PCR) was performed to verify the integrity and viability of pathogens after PUV treatment. The extracted DNA from *E. coli*, *S. aureus*, *B. cereus*, *C. albicans* and *S. typhimurium* were thawed, and 1 ng was mixed with the master mix according to Table 4. Bacterial 16S rDNA was amplified with the primers Pro341F and Pro805R (Table 5), with an expected product of 464 bp (Takahashi et al. 2014). For *C. albicans*, 18S rDNA primers SS5F and SS3R were used, and a product of 1800 bp was expected from amplification (Matsumoto et al. 2010).

**Table 4:** PCR reagents and cycling conditions

	Molarity	Cycling conditions	Molarity	Cycling conditions
5X Platinum Buffer	1X	94 °C – 2’ *94 °C – 15” *60 °C – 15” *68 °C – 15” *25 cycles	1X	95 °C – 1’ *95 °C – 15” *55 °C – 15” *72 °C – 30” 72 °C – 7’ *25 cycles
2 mM dNTP Mix	0.2 μM		0.2 μM	
Forward primer	0.2 μM		0.265	
Reverse primer	0.2 μM		0.265	
Taq polymerase	0.04 U/μL		0.0275	
Water	-		-	
Genomic DNA	1 ng		1 ng

**Table 5:** Primer sequence and expected product in base pairs (bp)

Primers	Sequence	Product (bp)
Pro341F	AATGATACGGCGACCACCGAGATCTACACTCTTTCCCTACACGACGCTCTTCCGATCTCCTACGGGAGGCAGCAGCCTACGGGNBGCASCAG	464
Pro805R	CAAGCAGAAGACGGCATACGAGATNNNNNNGTGACTGGAGTTCAGACGTGTGCTCTTCCGATCTGACTACNVGGGTATCTAATCC	
SS5F	GGTGATCCTGCCAGTAGTCATATGCTTG	1800
SS3R	GATCCTTCCGCAGGTTCACCTACGGAAACC	

PCR products were loaded with agarose 1X onto 1.5% agarose gel stained with SyBR green. Amplicons were visualized in a UV chamber, and band pixels were quantified on the software ImageJ (Rasband, 2018) to estimate the cDNA concentration.

### Statistical analysis

Results were analysed on the *Statistica* software version 10 (Statsoft, USA) by Student’s *t*-test comparing means, and *p* < 0.05 was considered significant. All experiments were done at least three times in triplicates.

## Results and discussion

The objective of this paper was to construct and test a small prototype pulsed ultraviolet (PUV) light system to demonstrate how this method might assist the shellfish industry in the depuration processes. The analysis of treatment parameters such as voltage, number of pulses and duration of treatment was performed to ensure a high reduction in contaminant levels. The recirculation system was based on current depuration plants in the shellfish industry where animals like oysters, for example, are kept in recirculating water treated by stationary UV systems to reduce the level of the pathogens purged from the oysters. The initial concentration of pathogens in treatment tanks ranged from 10^5^ to 10^8^ CFU/mL, simulating a highly contaminated environment. These levels are not usually found in seawater; however, the parameters were chosen to test the robustness and efficacy of PUV treatment in critical conditions, for example, a high level of bacteria can interfere with UV penetration depth. The preliminary tests to identify the voltage, number of pulses and duration of treatment were done to ensure high reduction and avoid excessive wear and tear of the PUV system.

The results from PUV treatment are presented in Fig. 4. Treatments 1 and 2, at 500 and 1000 V for 1 min, respectively, were the shortest and had fewer pulses (Table 1), resulting in microbe cell viability reductions ranging from 0.08 log_10_ for *E. coli* to 1.04 log_10_ for *B. cereus* (Fig. 4c) compared to the level in not treated (NT). The cell viability reduction (CVR) was not statistically significant between treatments 1 and 2 (Fig. 4a–e), showing the need for a prolonged treatment for improvement. Treatment 3 was prolonged to 10 min and the PUV operated at 500 V; however, reductions remained between 0 log_10_ for *B. cereus* and 0.79 log_10_ for *E. coli* compared to NT. A major increase in lethality was only observed when 1000 V pulses were applied for 10 min on treatment 4 (Fig. 4a), reducing the level of *E. coli* by 2.62 log_10_*.* The relation between the number of pulses, duration and voltage was also observed for treatments 5 and 6 with a total of 60 pulses per minute applied in each treatment. Considering the results of all pathogens, lower CVR reductions from 2.97 log_10_ to 4.12 log_10_ were obtained by applying 60 pulses per minute and 500 V when compared to the PUV unit operating at 1000 V and 60 pulses per minute, with a CVR from 3.32 log_10_ to 4.42 log_10_. This fact is explained by the higher energy and UV output of 1000 V pulses delivering 20 J and 12.9 J/cm^2^, respectively, while 5 J and 3.2 J/cm^2^ were delivered by 500 V pulses (Tables 1 and 2). CVR averages were statistically lower with 1000 V treatment than with 500 V for *S. aureus* (*p* = 0.0019), *B. cereus* (*p* = 0.0377) and *S. typhimurium* (*p* = 0.0036). Therefore, the conditions of treatment 6 were chosen, and 1000 V, 60 pulses/min and 10 min employed in further assays. In terms of pathogen resistance to PUV treatment, the bacterium *E. coli* was the most affected. Reductions ranged from 0.08 (T1) to 4.42 log_10_ (T6) followed by *S. aureus* from 0.46 (T2) to 4.41 log_10_ (T6), *S. typhimurium* from 0.15 (T1) to 4.19 (T6), *C. albicans* from 0.02 (T2) to 4.09 log_10_ (T6) and *B. cereus* from 0 (T3) to 3.32 (T6). Therefore, the highest CVR reductions were observed for all pathogens when 1000 V, 60 pulses and 10 min treatment were applied.

The optimal conditions for the PUV treatment were verified, and CVR is presented in Fig. 4f. All CVR were statistically significant; the highest observed was 5.63 log_10_ for *S. aureus*, followed by 5.15 log_10_ for *C. albicans*, 5 log_10_ for *S. typhimurium*, 4.59 log_10_ for *B. cereus* and 4.55 log_10_ for *E. coli*. These results are in accordance with the same treatment tested in preliminary assays as average CVR were not statistically different (*p* = 0.1044).

**Fig. 5:**
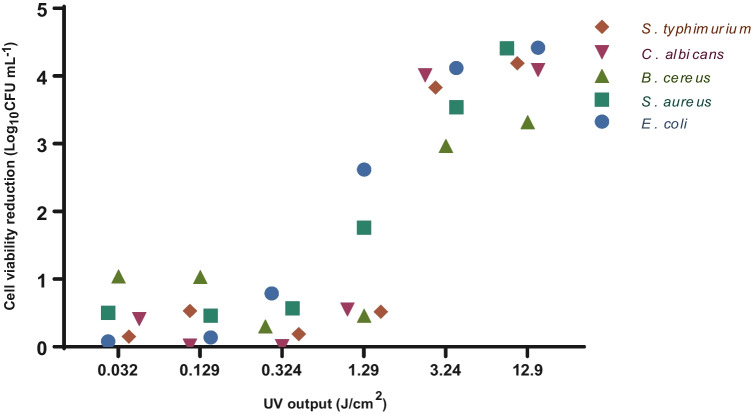
Cell viability reduction of *E. coli*, *S. aureus*, *B. cereus*, *C. albicans* and *S. typhimurium* by UV output of 0.032 J/cm^*2*^ for treatment 1, 0.129 J/cm^*2*^ for treatment 2, 0.324 J/cm^*2*^ for treatment 3, 1.29 J/cm^*2*^ for treatment 4, 3.24 J/cm^*2*^ for treatment 5 and 12.9 J/cm^*2*^ for treatment 6

Log reduction of cell viability by UV output of treatments 1 to 6 is presented in Fig. 5. A UV output between 0.032 and 0.324 J/cm^2^ resulted in the reduction of cell viability of approximately 1 log_10_ to all tested strains. *E. coli* and *S. aureus* were more sensitive to a UV output of 1.29 J/cm^2^ and presented a 1.76 and 2.62 log_10_ reduction, respectively (Fig. 5). To achieve reductions from 3 to 4, a UV output from 3.24 to 12.9 J/cm^2^ was required for all strains.

To ensure bivalve safety for consumers, alternative depuration methods and immune system stimulation approaches have been explored. A biological approach was reported by Jun et al. (2014) applying bacteriophage against *V. parahaemolyticus* in artificially contaminated oysters. Bacterial growth inhibition of 7.4 × 10^5^ CFU/mL after 12 h of phage application was reported. Probiotic bacteria can also be used to facilitate the depuration process and stimulate the shellfish immune system. Fajardo et al. (2014) obtained an isolate from a total of 365 bacteria from the shellfish digestive gland with in vitro antibacterial and antiviral activities. Ahmadi et al. (2015) reported a bio-physical method applying bacteriophages and high hydrostatic pressure for the inactivation of *S. flexneri* and *V. cholerae* in salmon and mussels. Complete inactivation of pathogens was achieved at 550 MPa for 5 min followed by the addition of a bacteriophage suspension at 10^9^ PFU/mL.

The treatment system described in this study was developed to be easily installable at current depuration plants without the need for modifications. The true challenge for such complex alternatives such as those already mentioned (biological and bio-physical) emerges when upscaling. For a flow rate of 10 mL/s (36 L treated per hour) and 0.2 L of water, a real scale depuration tank of 500 L would require 14 h of operation. The usual levels of contaminants are approximately 10^1^–10^3^ CFU/mL, reaching 10^4^ in class C areas, much lower than the 10^8–9^ CFU/mL tested here, and would require a shorter period of treatment. Furthermore, additional PUV chambers can be set together in parallel to increase the depuration efficiency and also get benefited by operating with the current low-pressure UV system installed in most of the depuration plants. UV efficacy was reported by Garcia et al. (2015) with a 99% reduction of recombinant adenovirus and murine norovirus levels after 24 h of treatment by a low-pressure UV lamp and UV output of 6.4 J/cm^2^. Low-pressure efficacy in *S. aureus* and *E. coli* was also observed by Fitzhenry et al. (2021) with 5.3 and 6 log_10_ and 14 mJ/cm^2^. The economics of running costs and capital equipment compared with UV have yet to be calculated accurately; however, pilot PUV systems will cost more until full optimization is made. PUV can be considered as a bolt-on system for depuration for the shellfish in terms of sharing cooperative functionality. Shutdown of a processing plant and relocation of shellfish result in significant financial losses, affecting logistics and deliveries, requiring a re-validation of the depuration facility which takes weeks to complete, and massive damages to the reputation of the shellfish industry sector, causing foodborne infections in local and abroad consumers. PUV can be employed in terms of risk mitigation and food security.

The results of DNA integrity assessed qualitatively by gel electrophoresis and semi-quantitatively by analysing band pixels are presented in Table 6. No amplification was observed in *S. aureus*, *C. albicans* and *S. typhimurium*. We believe that PUV treatment has affected the availability and integrity of DNA while the same amount of template was used for PCR and quantified in high-accuracy fluorometer Qubit (Invitrogen), and it did not amplify. The disruptive and genotoxic effects of UV light are widely described. UV light induces the photoreactive potential of purines (adenine and guanine) and pyrimidines (cytosine, thymine and uracil) in DNA, triggering the formation of mutagenic DNA lesions and inactivating the replication, leading to a reduction in CVR. Oxidative stress is also observed in cells exposed to UV, leading to produce reactive oxygen species (ROS) that attack nucleotide pools and stress-mediated mutations (Ikehata and Ono, 2011).

**Table 6:** Average peak area of amplicons bands from PCR of *E. coli*, *S. aureus*, *B. cereus*, *C. albicans* and S. typhimurium

	Pathogen	Average peak area		Ratio
		Non-treated	Treated	
1	*E. coli*	10,492.42	1692.26	6.2
2	*S. aureus*	10,111.01	0.00	-
3	*B. cereus*	11,770.74	796.48	14.78
4	*C. albicans*	12,607.45	0.00	-
5	*S. typhimurium*	8431.78	0.00	-

Pullerits et al. (2020) investigated the effect of UV doses of 250, 400 and 600 J/m^2^ on bacterial communities in water from a water treatment plant treated in a low-pressure UV system consisting of 10 rows of four UV lamps. The authors identified a long-term effect of UV irradiation that continued influencing the microbial dynamics after treatment and amplicons with greater guanine/cytosine contents were more resistant to UV treatment. In this study, *E. coli* and *B. cereus* had significantly lower amplification rates of 6.2 and 14.78-fold reduction, respectively. It was not possible to correlate; however, the lower amplification with lower CVR shown in Fig. 4 as the same amount of DNA to PCR was used for all pathogens and resulted in amplifications similar to those observed in non-treated samples (Table 6).

*B. cereus* is Gram-positive spore-forming bacterium widely distributed in nature and has been frequently reported to be the causative agent of food poisoning (FSAI, 2016; Rowan, 2019). *Bacillus* endospores are tolerant of environmental stresses and are often used as bioindicator organisms for evaluating the efficacy of disinfection and sterilization modalities. Garvey and Rowan (2015) previously reported that pulsed UV can inactivate 1.5 log_10_ *B. cereus* and *B. megaterium* endospores in a flow-through system using a UV dose of 21.6 μJ/cm^2^ and 6.46 μJ/cm^2^, respectively. A spore, *B. cereus* and its toxins can be heat resistant, requiring an extended pre-cooking procedure to reduce it to safe levels. Its resistance was observed by gel electrophoresis and detection of amplification bands post-PUV treatment and reduction in 4.59 log of *B. cereus* cells in a 10-min treatment. The PUV treatment method must be modified to better address the contaminant resistance. Taylor et al. (2020), for example, reported higher reductions of *B. cereus* spores when narrowing the UV to 222 nm, a peak also emitted in the present PUV protocol (Fig. 2). *E. coli* is a Gram-negative bacterium that is frequently associated with causing human and animal infections and can harbour multiple antibiotic resistance (AMR) genes. Zhang et al. (2017) reported its resistance when assessing the effect of a low-pressure UV treatment on antibiotic-resistant *E. coli* (AREC) isolated from a wastewater treatment plant. AREC required a higher UV dose of 20 mJ/cm^2^ to cause reduction when compared to antibiotic-sensitive strains (8 mJ/cm^2^).

The periodical monitoring of seawater and farm areas is important for safeguarding and for helping consumers helping the industry in decision-making (Fehrenbach et al. 2022). For shellfish producers, the water quality in these areas has been threatened by the expansion of cities and agriculture to shoreline. Irregular discharges of leachates and urban effluents represent a direct source of faecal contamination, mainly monitored and detected by *E. coli* levels, and this is a parameter considered when assessing the need for post-harvest manipulation. Other contaminants can also be present as chemicals and biotoxins that must also be periodically analysed. The location and boundaries of production areas are classified according to the level of faecal contamination. Regulation (EC) No. 854/2004 establishes the requirements for production areas in the European Union. Class A areas have the lowest risks and can be collected for direct human consumption if in accordance with health standards for live bivalves. Classes B and C cannot exceed 4600 and 46,000 *E. coli* per 100 g of flesh and intravalvular liquid, respectively, in the most probable number (MPN) test (EC, 2019). Classes B and C must be depurated at purification facilities to reduce the level of contaminants. For example, guidance for local action on handling high *E. coli* results, pollution events and biotoxin results was released by Food Standards Agency (FSA) – England and Wales (2021). Levels of *E. coli* above 700, 18,000 and 46,000 per 100 g of shellfish for classes A, B and C, respectively, will require immediate action, from downgrading to temporary closure (FSA, 2021).

**Fig. 6:**
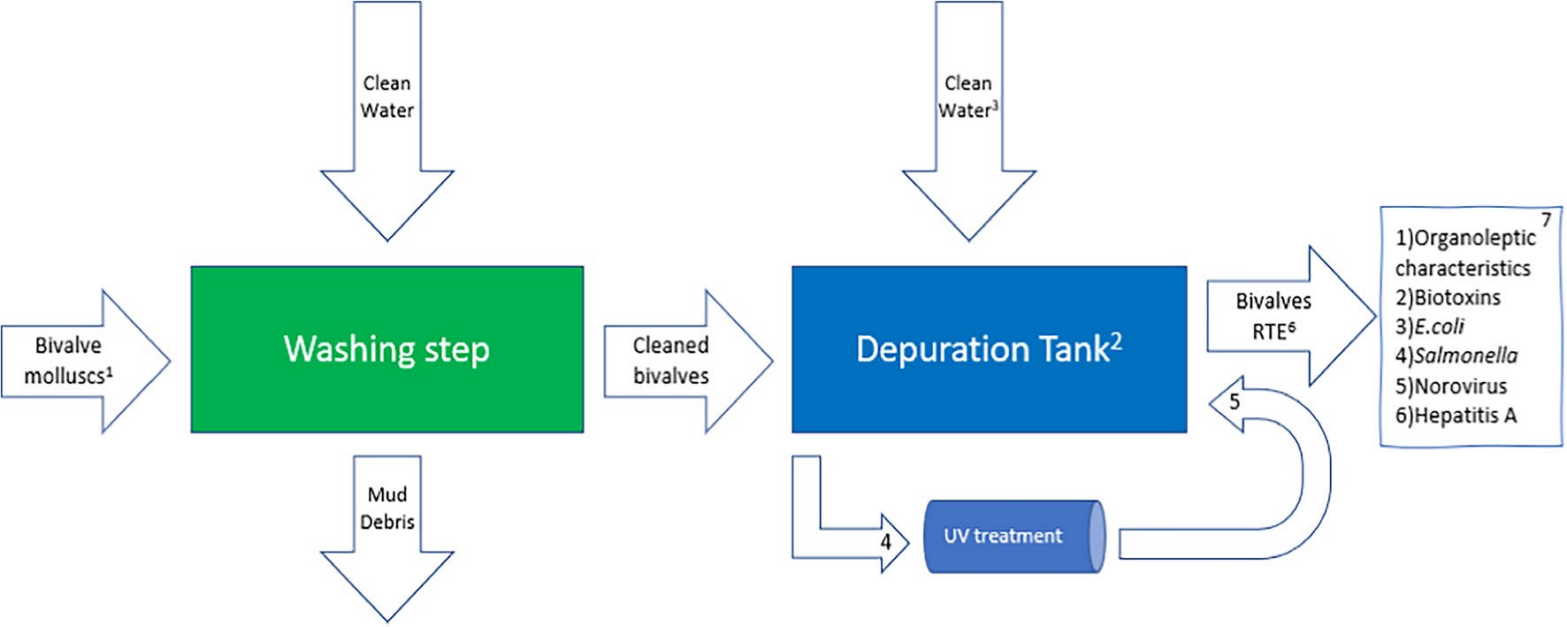
Usual depuration system for bivalves

The usual system employed at purification facilities for bivalves is presented in Fig. 6. It consists of two main steps of washing and depuration. First, bivalves are transported by conveyor to a cleaning facility where they are graded according to size and serially cleaned in washing equipment. Then, the bivalves are placed into depuration tanks with clean water that recirculates in a purification system equipped with a UV system for disinfection. This system is usually effective for low contamination levels. The development of new alternatives is necessary to ensure efficient depuration, especially during winter when contaminants are higher and norovirus, for example, can reach 20,000 genome copies g^−1^ of digestive tissue (Rajko-Nenow et al. 2012). When this occurs, bivalves must be depurated for longer periods and/or also combined with higher water temperatures which can overload the facility period. Alternatively, they can be reintroduced into areas of cleaner water (EC, 2004; FSAI et al. 2018). These alternatives not only increase the production costs but also affect the production chain, possibly leading to delivery delays and affecting quality decrease to stressing of shellfish as well as increasing costs to the consumer. PUV treatment reached a bacterial removal of 5.63 log in highly contaminated artificial seawater after 10 min of treatment and could potentially support the removal of other contaminants such as NOV. It also supports the depuration of shellfish cultured in highly contaminated waters such as class C, where *E. coli* levels can reach 46,000 MPU per 100 g of flesh and intravalvular liquid (EC, 2019). Considering 46,000 MPU per 100 g as the *E. coli* load in shellfish, the PUV treatment can reduce it to safe consumption levels < 230 *E. coli*/100 g. However, it is necessary to consider the required incubation period for shellfish to depurate and shellfish load per batch. Figure 6 presents the usual depuration system for bivalves: (1) bivalves harvested from class B and C production areas or a purification centre or another dispatch centre. Production areas are classified according to the level of contamination, where A is the least and C the most contaminated; (2) bivalves must be kept at stressless conditions that support the filter-feeding act; (3) clean seawater collected and analysed before usage, it can be treated for safety measures; (4) water from depuration tanks is pumped through a UV chamber to inactivate pathogens; (5) treated water returns to depuration tank; (6) bivalves clean and ready-to-eat (RTE) are immediately packed and kept at 4 °C; and (7) parameters that must be verified in the bivalves before commercialization.

In this research paper, we present a PUV system of broad efficacy reducing in 4.5–5.6 log_10_ the initial level of 5 common foodborne pathogens of different complexity and persistence. PUV treatment is a promising alternative to assist in the reduction of pathogen levels at depuration plants. Other pathogens, such as *Vibrio* sp. and norovirus, will also be tested in future studies to assess the PUV efficacy. The high disinfection efficiency and short treatment duration support the upscaling of PUV at the pilot-scale level, as well as the modelling of the treatment.

## Conclusions

- The main parameters for PUV treatment such as voltage, number of pulses and duration successfully led to higher reductions in cell viability, as verified by posterior treatment.
- A UV output of 12.9 J/cm^2^ at 1000 V, 60 pulses/min for 10 min of treatment reduced the levels of common foodborne pathogens to a maximum of 5.63 log_10_ compared to no treatment.
- PUV treatment disrupted the pathogen DNA where *S. aureus*, *C. albicans* and *S. typhimurium* were not amplified by PCR; *E. coli* and *B. cereus* bands were detected by the software ImageJ, however, reduced by 6.2- to 14.8-fold compared to before treatment.
- Excellent depuration results were achieved with the same treatment conditions for all tested microorganisms without requiring specific UV conditions.
- The efficient depuration of different pathogens and simple set-up of the PUV system favours future testing at the pilot scale.

## Data Availability

Data and materials for this study are available and will be provided to the journal upon request.

## Appendix

See Table 7.

**Table 7:** Spectrum output (μJ/cm^2^) at different distance from the flash lamp (cm) and bandwidth wavelengths

Distance (cm)	< 300 nm	300–400 nm	400–500 nm	500–600 nm	600–700 nm	> 700 nm
10			630	370	444	1877
15	346	222	295	156	191	778
20	166	140	168	93	112	462
25	129	84	114	67	73	302
30	76	57	83	42	54	215
35	62	43	57	38	40	158
40	40	39	48	26	30	122
45	38	27	40	20	24	98
50	34	21	33	16	20	79
